# Influence of Race and High Laminar Shear Stress on TNFR1 Signaling in Endothelial Cells

**DOI:** 10.3390/ijms241914723

**Published:** 2023-09-29

**Authors:** Maitha Aldokhayyil, Dulce H. Gomez, Marc D. Cook, Andreas N. Kavazis, Michael D. Roberts, Thangiah Geetha, Michael D. Brown

**Affiliations:** 1 School of Kinesiology, Auburn University, Auburn, AL 36849, USA; 2 Department of Physical Therapy, College of Applied Medical Sciences, Imam Abdulrahman Bin Faisal University, P.O. Box 1982, Dammam 31441, Saudi Arabia; 3 Department of Kinesiology, North Carolina Agriculture and Technology State University, Greensboro, NC 27411, USA; 4 Department of Nutritional Sciences, College of Human Sciences, Auburn University, Auburn, AL 36849, USA

**Keywords:** endothelial dysfunction, racial differences, laminar shear stress, tumor necrosis factor receptor1, atherosclerosis, African Americans

## Abstract

Tumor necrosis factor (TNF) binding to endothelial TNF receptor-I (TNFR-I) facilitates monocyte recruitment and chronic inflammation, leading to the development of atherosclerosis. In vitro data show a heightened inflammatory response and atherogenic potential in endothelial cells (ECs) from African American (AA) donors. High laminar shear stress (HSS) can mitigate some aspects of racial differences in endothelial function at the cellular level. We examined possible racial differences in TNF-induced monocyte adhesion and TNFR1 signaling complex expression/activity, along with the effects of HSS. Tohoku Hospital Pediatrics-1 (THP-1) monocytes were used in a co-culture system with human umbilical vein ECs (HUVECs) from Caucasian American (CA) and AA donors to examine racial differences in monocyte adhesion. An in vitro exercise mimetic model was applied to investigate the potential modulatory effect of HSS. THP-1 adherence to ECs and TNF-induced nuclear factor kappa B (NF-κB) DNA binding were elevated in AA ECs compared to CA ECs, but not significantly. We report no significant racial differences in the expression of the TNFR-I signaling complex. Application of HSS significantly increased the expression and shedding of TNFR-I and the expression of TRAF3, and decreased the expression of TRAF5 in both groups. Our data does not support TNF-induced NF-κB activation as a potential mediator of racial disparity in this model. Other pathways and associated factors activated by the TNFR1 signaling complex are recommended targets for future research.

## 1. Introduction

Tumor necrosis factor (TNF) is a potent, pro-inflammatory cytokine produced predominately by activated leukocytes. One of the main targets of TNF is the endothelium, where it initiates a cascade of events leading to endothelial dysfunction [1,2,3,4]. The diverse effects of TNF are mediated primarily by the cell surface receptor TNF receptor 1 (TNFR1) [5]. Once activated by TNF binding, TNFR1 forms a signaling complex with adaptor proteins and TNFR-associated factors (TRAFs) and activates multiple signaling pathways like nuclear factor kappa B (NF-κB) and mitogen-activated protein (MAP) kinase [6,7,8,9]. This induces the expression of adhesion molecules and chemokines that cause monocyte recruitment and activation, consequently amplifying inflammation and initiating atherogenesis [10,11,12,13]. Atherosclerotic arteries exhibit greater levels of all TRAFs compared to control arteries, as TRAFs have a role in plaque progression [14]. Additionally, TNF has a detrimental effect on endothelial nitric oxide synthase (eNOS) promoter activity, compromising nitric oxide (NO) bioavailability, a hallmark of endothelial dysfunction [15].

The efficacy of exercise training in improving endothelial function by increasing NO bioavailability and reducing inflammation is well established [16,17,18]. Further, soluble TNFR1 (sTNFR1) levels improve with aerobic training [19,20,21]. TNFα-converting enzyme (TACE) mediates the shedding of the TNFR1 extracellular domain, forming sTNFR1 in response to inflammatory stimuli, thereby decreasing the expression of active membrane receptors [1,5,22]. sTNFR1 binds to circulating TNF and neutralizes its activity [22]. Clinically, administering sTNFR1 is an effective treatment strategy to control inflammatory conditions [5].

High laminar shear stress (HSS) mediates functional and structural vascular adaptations associated with exercise training [23]. Evidence suggests that HSS mitigates inflammation and induces atheroprotective effects [24]. In vitro, the application of HSS is a validated exercise mimetic model. Specifically, HSS has been shown to inhibit some of the TNF-induced downstream effects, such as MAP kinases, particularly c-Jun N-terminal kinases (JNK) [24,25,26]. Moreover, HSS upregulates TRAF3 expression in HUVECs, leading to suppressed CD40-induced AP-1 activation [26]. Thus, TRAF3 is a possible mechanosensitive mediator of the HSS atheroprotective effect. Additionally, HSS has been shown to induce a greater reduction in oxidative stress and inflammatory markers in African American (AA) endothelial cells (ECs) compared to Caucasian American (CA) ECs [27].

AA individuals have the highest prevalence of cardiovascular disease (CVD) [28]. Recent data from the Multi-Ethnic Study of Atherosclerosis (MESA) have demonstrated that AA individuals had the highest common carotid intima-media thickness (cIMT), which is used as a measure of subclinical atherosclerosis [28]. This may be partially attributed to the prevalence of endothelial dysfunction in this population, manifested by lower flow-mediated dilation (FMD), thicker cIMT, and higher endothelin-1 levels (ET-1), a potent constrictor, compared to CA counterparts [29]. Even young and healthy AA have lower FMD levels, a measure of endothelial function, than CA [30,31]. Additionally, our previous in vitro data showed that AA ECs exhibit greater oxidative stress and heightened inflammation compared to CA ECs [27]. AA human umbilical vein ECs (HUVECs) express higher levels of NADPH oxidase subunits and lower superoxide dismutase 1 (SOD1) activity than CA HUVECs [32]. Moreover, we showed that AA HUVECs produce higher basal levels of interleukin (IL)-6, an inflammatory cytokine, than CA HUVECs in response to TNF stimulation [33].

Racial differences in EC responses to stimuli, particularly TNF, could play an important role in the promotion of endothelial dysfunction, plaque development, and consequent CVD. Despite the compelling data supporting a higher prevalence of endothelial dysfunction and CVD in AA, it is predominantly of an observational nature [30,31,34,35,36]. Evidence explaining the mechanism(s) prompting this disparity is still lacking. Therefore, research exploring the underlying cellular mechanism(s) that can be targeted for treatment/prevention is needed and of high importance.

In this in vitro study, we hypothesized that AA-derived ECs would exhibit greater THP-1 monocyte adhesion and higher expression/activity of the TNFR1 signaling complex that can be attenuated by HSS. Using naïve ECs such as HUVECs, a valid model to mechanistically investigate EC function, minimizes the influence of pre-existing factors and enables race/ethnicity-related EC profiling. The aims of our study were to examine the following: (1) racial differences in TNF-induced monocyte adhesion; (2) racial differences in TNFR1 signaling complex expression/activity, TNFR1 shedding, and downstream effects; and (3) the effects of HSS on mitigating expected racial differences.

## 2. Results

### 2.1. THP-1 Monocyte Adhesion

**Figure 1:**
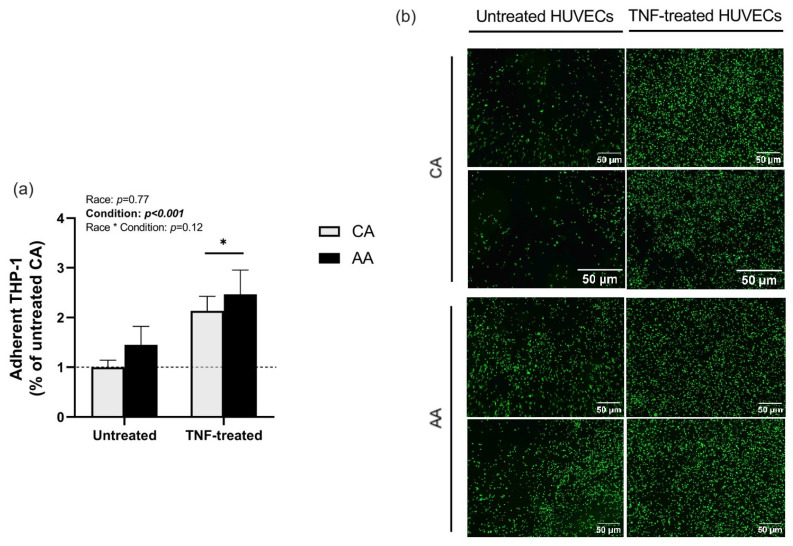
THP-1 monocyte adhesion on HUVECs. (**a**) Adhesion of labeled THP-1 to TNF-treated HUVECs was quantified by fluorescence microplate reader. Net fluorescence is expressed relative to CA untreated HUVECs. (**b**) Representative images of labeled THP-1 adhesion on untreated and TNF-treated HUVECs. Scale bar, 50 μm. Data are represented as mean ± SE from three independent experiments in three different cell lines per racial group. * *p* < 0.001 compared to untreated.

TNF treatment increased THP-1 monocyte adhesion in both racial groups (*p* < 0.001, Figure 1). However, no racial (*p* = 0.77, Figure 1) or interaction effects (*p* = 0.12, Figure 1) were detected.

### 2.2. TNFR1 Signaling Complex and HSS

**Figure 2:**
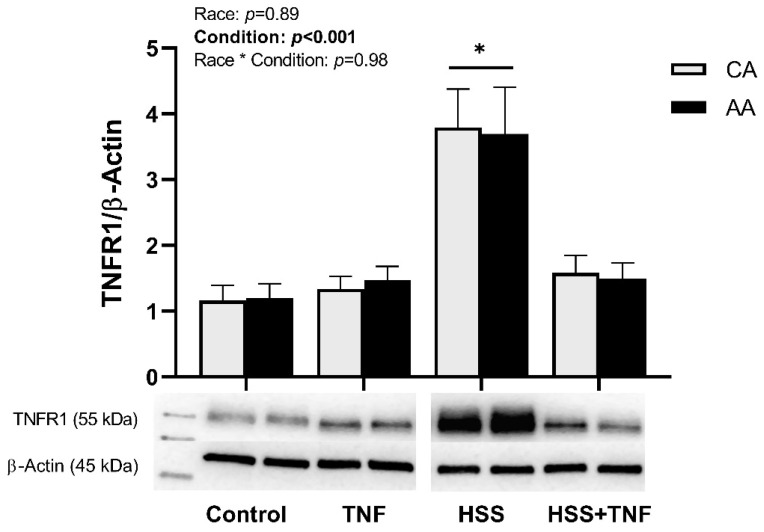
HSS upregulates TNFR1 expression in CA and AA HUVECs. CA and AA HUVECs were incubated with TNF (30 ng/mL, 6 h), exposed to HSS (20 dyne/cm^2^, 24 h), or HSS (20 dyne/cm^2^, 24 h) followed by TNF (30 ng/mL, 6 h). HSS significantly increased TNFR1 expression in both groups. Densitometric quantification was normalized to housekeeping protein (β-actin). Data are represented as mean ± SE from three independent experiments in three different cell lines per racial group. * *p* < 0.001 compared to other conditions.

**Figure 3:**
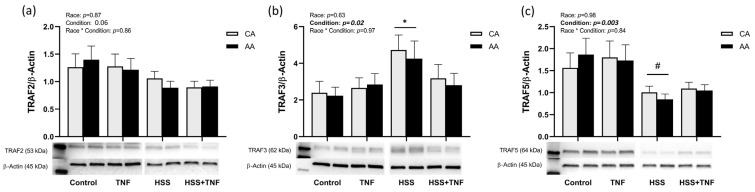
TRAF expression under different experimental conditions. CA and AA HUVECs were incubated with TNF (30 ng/mL, 6 h), exposed to HSS (20 dyne/cm^2^, 24 h), or HSS (20 dyne/cm^2^, 24 h) followed by TNF (30 ng/mL, 6 h). HSS significantly upregulated TRAF3 expression (**b**) and downregulated TRAF5 (**c**). However, TRAF2 expression did not change across all conditions (**a**). Data are represented as mean ± SE from three independent experiments in three different cell lines per racial group. * *p* = 0.02 compared to other conditions; # *p* = 0.003 compared to control and TNF.

TNFR1 and TRAF3 were significantly upregulated in response to HSS compared to all other conditions (*p* < 0.001, Figure 2 and *p* = 0.02, Figure 3b), whereas TRAF5 was downregulated in response to HSS, an effect that was not altered by TNF treatment (*p* = 0.003, Figure 3c). TRAF2 levels were not significantly affected by any of the experimental conditions (*p* = 0.06, Figure 3a). No racial or interaction effects in the expression of TNFR1 or TRAFs were detected.

### 2.3. TNFR1 Shedding and HSS

**Figure 4:**
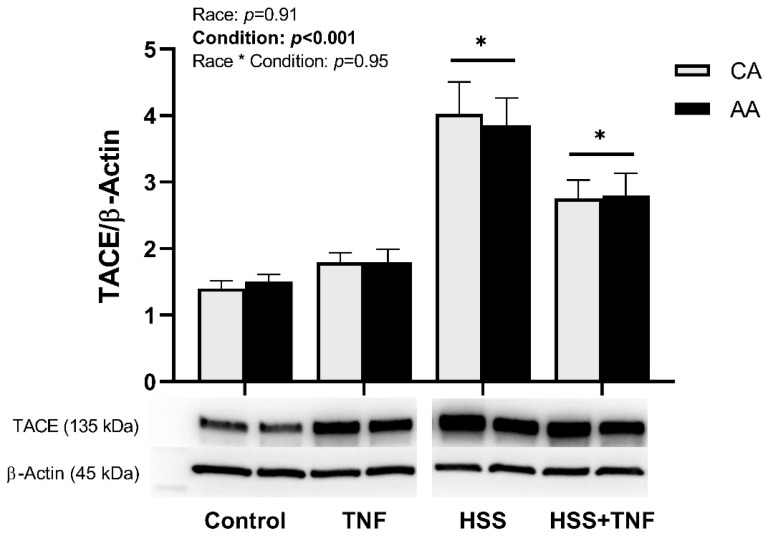
HSS upregulates TACE expression in CA and AA HUVECs. CA and AA HUVECs were incubated with TNF (30 ng/mL, 6 h), exposed to HSS (20 dyne/cm^2^, 24 h), or HSS (20 dyne/cm^2^, 24 h) followed by TNF (30 ng/mL, 6 h). Densitometric quantification was normalized to housekeeping protein (β-actin). Data are represented as mean ± SE from three independent experiments in three different cell lines per racial group. * *p* < 0.001 compared to control and TNF.

**Figure 5:**
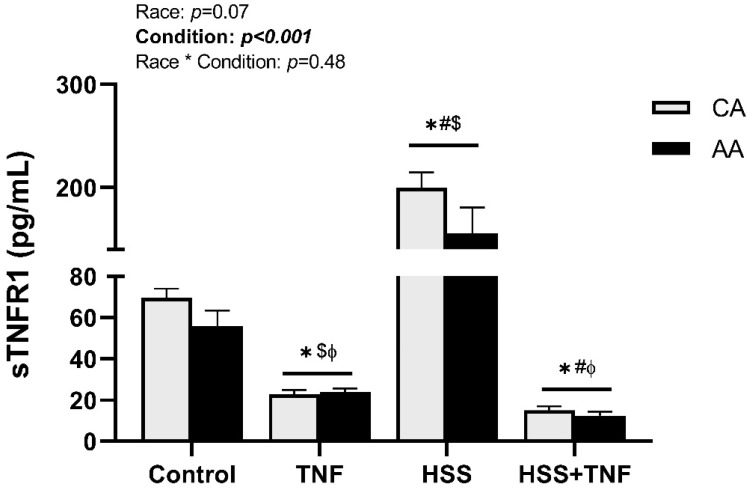
TNFR1 shedding under different experimental conditions. CA and AA HUVECs were incubated with TNF (30 ng/mL, 6 h), exposed to HSS (20 dyne/cm^2^, 24 h), or HSS (20 dyne/cm^2^, 24 h) followed by TNF (30 ng/mL, 6 h). Shedding of TNFR1 was quantified in cell culture supernatant. High shear stress-induced upregulation of sTNFR1 was significantly suppressed by TNF treatment. Data are represented as mean ± SE from three independent experiments in three different cell lines per racial group. * *p* < 0.001 compared to control; # *p* < 0.001 compared to TNF; ɸ *p* < 0.001 compared to HSS; $ *p* < 0.001 compared to HSS + TNF.

HSS induced an upregulation of TACE expression in both racial groups. TACE expression remained significantly elevated after the follow-up TNF treatment, but it was numerically lower compared to the HSS group (*p* < 0.001, Figure 4). Further, HSS significantly increased sTNFR1 levels in CA and AA HUVECs, an increase that was diminished by the follow-up TNF treatment (*p* < 0.001, Figure 5). Shedding of TNFR1 was lower in AA HUVECs compared to CA HUVECs; however, this difference was not statistically significant (*p* = 0.07, Figure 5). No interaction effect on the expression of TACE or TNFR1 shedding was detected.

### 2.4. NF-κB Binding Activity

**Figure 6:**
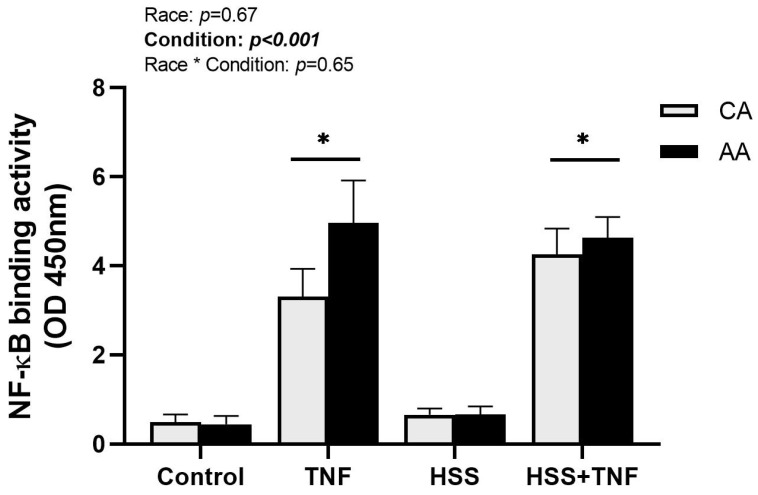
NF-κB binding activity under different experimental conditions. CA and AA HUVECs were incubated with TNF (30 ng/mL, 6 h), exposed to HSS (20 dyne/cm^2^, 24 h), or HSS (20 dyne/cm^2^, 24 h) followed by TNF (30 ng/mL, 6 h). NF-κB binding activity was quantified in nuclear extract. TNF-induced NF-κB activation was not altered by HSS in both groups. Data are represented as mean ± SE from three independent experiments in three different cell lines per racial group. * *p* < 0.001 compared to control and HSS.

TNF treatment significantly increased NF-κB DNA binding activity in both racial groups (*p* < 0.001, Figure 6). NF-κB DNA binding activity was numerically higher in AA HUVECs in response to TNF treatment; however, it was not significant (*p* = 0.67, Figure 6). Additionally, HSS had no protective effect against TNF-induced NF-κB activation (*p* < 0.001, Figure 6), and there was no interaction effect on NF-κB activation across all conditions (*p* = 0.65, Figure 6).

## 3. Discussion

**Figure 7:**
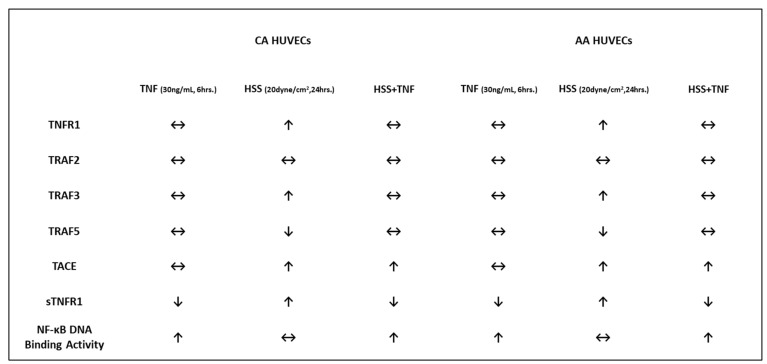
A summary diagram of the results. ↑ increased, ↓ decreased, ↔ unchanged.

Despite the slight elevations observed in baseline THP-1 adhesion and TNF-induced NF-κB DNA binding, we found no significant racial differences in (1) TNF-induced monocyte adhesion, (2) TNF-induced activation of the TNFR1 signaling complex, or (3) the modulatory effects of HSS on TNFR1 signaling. Additionally, HSS did not mitigate TNF effects on NF-κB binding activity, diminished shedding of TNFR1, and unexpectantly modulated TRAF5 protein expression (a novel finding in this study). Although our results do not support our proposed hypothesis of higher TNFR1 signaling complex expression/activity and downstream effects in AA-derived ECs, HSS-induced atheroprotective effects mitigated TNFR1 signaling in both racial groups. A summary of the results is illustrated in Figure 7.

TNFR1 activation by TNF binding induces endothelial dysfunction manifested by suppressed eNOS activity, higher oxidative stress levels, and amplified expression of adhesion molecules. Adhesion molecules (such as VCAM-1, ICAM-1, MCP-1, and E-selectin) play a key role in recruiting monocytes and initiating atherogenesis [2,11]. Plasma levels of TNF and adhesion molecules are associated with subclinical atherosclerosis [37]. Additionally, data from the Atherosclerosis Risk in Communities (ARIC) study highlighted adhesion molecules as independent molecular markers of coronary heart disease [38]. Previous research from our lab suggests a heightened inflammatory response to TNF in ECs from AA donors [33]. Further, basal and induced ICAM-1 and VCAM-1 levels are higher in AA HUVECs compared to CA HUVECs [39]. Therefore, we hypothesized TNF-induced monocyte adhesion to be higher in AA HUVECs; however, our results do not support this hypothesis.

Research implementing co-culture systems to investigate molecular mechanisms prompting racial differences in CVD is scarce. In our experiments, we used THP-1 cells, a validated monocyte cell model, to investigate atherogenesis and other vascular inflammatory conditions [40]. Nonetheless, we acknowledge the differences between THP-1 cells and primary monocytes and the limitations of THP-1 cells as a monocyte cell line model. Interestingly, recent evidence has shown that peripheral blood mononuclear cells (PBMCs) from young-normotensive AA exhibit higher resting oxidative stress levels [41]. Thus, examining the interactions between AA-derived PBMCs and AA-derived ECs would also be relevant.

Contrary to our hypotheses, our results do not support differential expression/activity of TNFR1 signaling between races. Both CA and AA HUVECs responded similarly to all experimental conditions. NF-κB activation was numerically higher in AA HUVECs in response to TNF; however, it was not statistically significant. This may be explained by two factors. First, we did not measure the expression or activity of MAP kinases or other transcription factors such as AP-1 that may play a bigger role in prompting racial differences than NF-κB signaling. Second, in addition to mediators of inflammation, NF-κB induces transcription of TNFR1 signaling negative regulators that may be differentially expressed, such as IκBα and A20. It has been shown that, despite the well-established suppressive effect of A20 on TNF-induced genes regulated by NF-κB, it does not interfere with the nuclear translocation of the NF-κB heterodimers or DNA binding [42]. If such is the case, we speculate lower expression/activity of negative regulators in AA ECs. Furthermore, HSS-induced shedding of TNFR1 tended to be lower in AA HUVECs; however, it was not statistically significant. Most available assays, including the one we have used, may not be sensitive enough to distinguish between the free form of sTNFR1 and that bound to TNF. Additionally, given that we did not measure TNF levels, we cannot speculate if sTNFR1 levels in AA HUVECs that are comparable to CA are sufficient to neutralize the potentially higher TNF levels in AA HUVECs.

Several clinical reports have demonstrated higher shedding of TNFR1 with aerobic exercise and its association with better vasodilation and functional outcomes [19,20,21,43]. In the present study, we used a cone and plate viscometer that mimics the shear stress patterns the endothelium is exposed to in vivo during aerobic exercise. Our results support clinical data in that HSS induces higher shedding of TNFR1. This was paralleled by a higher expression of TACE. An interesting finding of this study was the HSS-induced upregulation of TNFR1. This could be a compensatory mechanism due to the HSS-induced inhibition of the TRAF2-TNFR1 interaction [26] and the increased shedding of membrane-bound TNFR1. Unexpectedly, the application of HSS prior to TNF treatment did not lessen the unfavorable effects of TNF, specifically the sustained NF-κB binding activity. Yamawki et al. [26] have suggested that shear stress atheroprotective effects are mediated by inhibiting MAP kinase signaling in addition to TRAF2-TNFR1 interaction. They have shown that pre-exposing rabbit aortas to HSS significantly attenuated TNF-induced VCAM-1 expression by inhibiting MAP kinase signaling, while TNF-induced NF-κB activation remained unaffected. Collectively, these results suggest signaling pathway specificity in terms of the atheroprotective effects of HSS.

TRAFs are a family of intracellular adaptor proteins that function as signal transducers for the TNF receptor superfamily [44,45,46,47]. TRAF2 is the most ubiquitously expressed of the TRAF family, and it plays a major role in activating NF-κB and AP-1 transcription factors [45]. While TRAF3 forms a complex with TRAF2 and cIAP1/2 and negatively regulates non-canonical NFκ-B signaling [46,48,49], TRAF5 is comparable to TRAF2 in structure and function. Using TRAF2-deficient cells, the redundant role of TRAF5 in TNF-induced activation of NF-κB was highlighted. However, TRAF5 had no effect on JNK signaling activation [50]. In regard to the effect of HSS on TRAFs, our results are in agreement with Urbich et al. [51]. HSS induced an upregulation of TRAF3, while TRAF2 levels remained unaffected. TRAF3 is an important negative regulator of NF-κB-inducing kinase [46,48,49]. Consistent with the anti-inflammatory effects of HSS, TRAF3 upregulation, brought about by HSS in this study, has been associated with inhibition of CD40-induced endothelial cell activation [51]. On the other hand, while Urbich et al. [51] showed no effect of HSS on TRAF5 levels in HUVECs, our results demonstrated a significant decrease in the expression of TRAF5 following the application of HSS. This finding could be due to differences in the doses of shear stress. Whereas they exposed HUVECs to 15 dyne/cm^2^ of shear for 18 h, we used 20 dyne/cm^2^ for 24 h. In vivo data suggest a negative regulatory role for TRAF5, as knocking out TRAF5 accelerates atherosclerosis in animal models. Additionally, higher systemic levels of TRAF5 are associated with recovery in patients with coronary heart disease [52]. Therefore, as one of the atheroprotective effects of laminar shear, we expected TRAF5 levels to increase with HSS. Yet, TRAF5 levels decreased. Hence, it is plausible that the role of TRAF5 as a pro- or anti-inflammatory mediator is cell-specific.

### Limitations

Different concentrations of serum were necessary to use in our culture media for different conditions. Serum deprivation is recommended for prime cells when examining proinflammatory cytokine signaling. Therefore, we used serum-free culture medium for the static conditions and serum culture medium for the shear stress conditions. This is mainly because our cone and plate shear stress model is optimized for a limited range of medium viscosities. Nonetheless, we used only 2% fetal bovine serum, which is considered to be in the lower range [53]. Additionally, we used HUVECs as our endothelial cell model, which is a validated cell model in the field of vascular physiology. It has been shown that TNF-induced inflammatory cell adhesion in HUVECs is comparable to human umbilical arterial endothelial cells (HUAEC) and primary human coronary artery endothelial cells (HCAEC) [54]. HUVECs are often used by our group and others to investigate molecular mechanisms of vascular disease and, in particular, racial disparity in endothelial (dys)function [27,31,55,56]. Further, utilizing HUVECs, which are naïve cells, can facilitate the identification of endothelial phenotypes and activities that may predispose some racial groups, such as AA, to adverse vascular outcomes. However, we acknowledge its drawbacks; hence, our results should be interpreted in relation to the molecular pathways and not the physiologic response. We also agree with the remarks of Cook et al. and Robinson et al. [39,57] that shed light on the advantages and disadvantages of using commercially available HUVECs and how vendors offer limited access to information about the health and lifestyle of the mother that may lead to epigenetic and phenotypic changes of these ECs. However, this study has provided necessary insight into the effect of pro-inflammatory TNFR1 signaling and the interactions between HSS and race on TNF-induced NF-κB activity.

## 4. Materials and Methods

### 4.1. Cell Culture

#### 4.1.1. HUVECs

HUVECs were purchased from Lonza (Morristown, NJ, USA) and cultured in the endothelial basal medium-2 medium containing 2% fetal bovine serum and growth supplements and used between passages 6 and 7. Cells from three AA and three CA donors were grown in parallel, and the experiment was repeated three times. This experimental design has been validated in our previous work [28,29,33]. Cells were maintained at 37 °C in a 5% CO_2_ atmosphere and cultured in gelatin-coated 100 mm tissue culture dishes. When cells reached 80–90% confluency, they were washed with Hank’s Balanced Salt Solution (HBSS) buffer and serum starved for 2 h before stimulation or application of shear stress. A concentration of 30 ng/mL of TNF (cat. No. 210-TA-020/CF) from R&D Systems (Minneapolis, MN, USA) was used and incubated for 6 h. This dose was chosen based on preliminary experiments.

#### 4.1.2. THP-1

THP-1 monocytes (ATCC^®^ TIB202™, Manassas, VA, USA) were grown in RPMI 1640 medium supplemented with 10% fetal bovine serum (Gibco, Waltham, MA, USA), 1% penicillin/streptomycin (VWR, Radnor, PA, USA), and 2 mM/L GlutaMAX (Gibco). Cells were cultured in T-75 flasks and maintained at 37 °C in a 5% CO_2_ atmosphere.

### 4.2. Cell Adhesion Assay

HUVECs were seeded in gelatin-coated 24-well plates at a density of 5 × 10^4^ cells per well. Once confluent, HUVECs were washed with HBSS buffer, serum starved for 2 hrs, and then activated with TNF (30 ng/mL, 6 h) or left untreated. THP-1 monocytes were labeled with 5 µM of Calcein-AM (cat. no. 354216) from Corning (Corning, NY, USA) and suspended in serum-free RPMI 1640 medium. After activation of HUVECs, labeled THP-1 cells were added to the HUVEC monolayer (5 × 10^5^ per well) and incubated at 37 °C with 5% CO_2_ for 1 hr. Labeled THP-1 cells were added to empty wells as a background control. Nonadherent THP-1 monocytes were removed and washed with 1X phosphate-buffered saline (PBS) three times. Adherent cells were then lysed with 200 µL of RIPA lysis buffer. To quantify cell adhesion, fluorescence intensity was measured with a SpectraMax M2 plate reader (Molecular Devices, San Jose, CA, USA) at an excitation wavelength set at 494 nm and an emission wavelength of 517 nm. Cell adhesion was determined as a percentage of the control (untreated HUVECs).

### 4.3. Laminar Shear Stress

After 2 hrs of serum deprivation, confluent monolayers were exposed to unidirectional shear stress for 24 h with a rotating cone-in-plate instrument at a 0.5° angle, designed for 100 mm tissue culture dishes, as we have used previously [55]. Cone-and-plate experimental models have the advantage of inducing flow with a moving upper conical boundary and, thus, do not generate any pressure gradients that could alter cell function [58]. Two conditions were applied: HSS (20 dyne/cm^2^) and HSS (20 dyne/cm^2^) followed by TNF (30 ng/mL, 6 h) incubation.

### 4.4. Western Blotting

Cell lysate was fractionated using the Nuclear Extraction Kit (cat. No. ab113474, Abcam, Cambridge, MA, USA) following the manufacturer’s instructions with some modifications. Cytosolic extract was used for Western blotting. For each experimental condition, 10–15 μg of protein was loaded into wells, with the amount kept constant within each gel. All samples were run on duplicate gels. Proteins were separated using SDS-PAGE (4–15% TGX gels, Bio-Rad, Hercules, CA, USA) according to molecular weight and transferred to PVDF membranes, which were blocked with 5% non-fat dry milk (NFDM) for 1 h at room temperature. This was followed by incubation with primary antibodies of interest with gentle agitation overnight at 4–8 °C. Primary antibodies used were as follows: TNFR1 (cat. no. 3736), TACE (cat. no. 6978), TRAF2 (cat. no. 4724), TRAF3 (cat. no. 4729), and TRAF5 (cat. no. 41658) from Cell Signaling Technology (Beverly, MA, USA). β-actin (cat. no. 3700) from Cell Signaling Technology was used as a loading control. Protein was visualized by chemiluminescent detection using an HRP illumination substrate, Luminata Forte (EMD Millipore, Billerica, MA, USA). The UVP ChemiDoc-It2 imaging system (UVP, Upland, CA, USA) was used to image each PVDF membrane, and VisionWorks software (8.19) was used to quantify protein expression by band densitometry analysis. Densities of selected proteins were expressed relative to a loading control for normalization.

### 4.5. Assays

**Figure 8:**
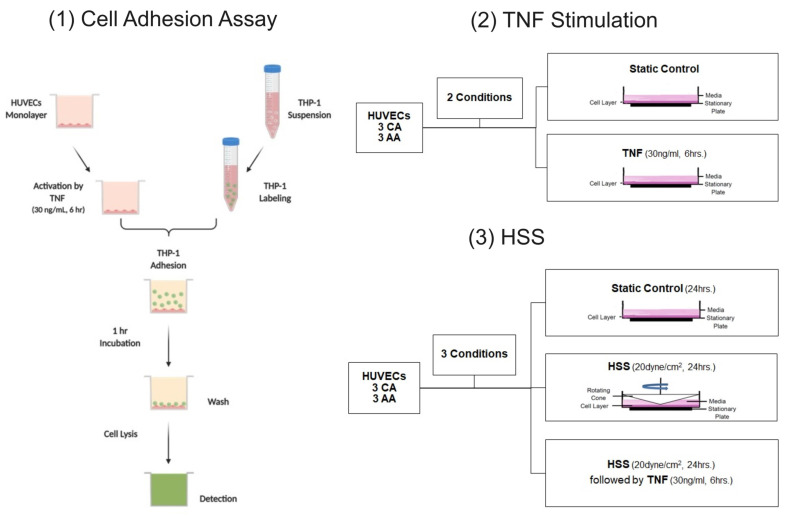
A flow chart of all experimental procedures. Created with BioRender.com (accessed on 19 September 2023).

Cell culture supernatant was collected, centrifuged, aliquoted, and immediately stored at −80 °C until analysis. The sTNFR1 assay kit (cat. no. DRT100) from R&D Systems (Minneapolis, MN, USA) was used to detect sTNFR1 production in HUVEC cell culture supernatant. The NF-κB factor assay kit (cat. no. 43296) from Active Motif (Carlsbad, CA, USA) was used to quantify NF-κB DNA binding activity in nuclear extracts. Figure 8 depicts a flow chart of all the experimental procedures.

### 4.6. Statistical Analysis

A two-way ANOVA was performed to examine any race-by-condition interaction effect on any of the outcome variables. Violations of assumptions were examined. Non-normally distributed data were natural log-transformed. Post hoc adjustment for multiple comparisons was done using Bonferroni’s test. Analysis was performed using SPSS version 26 (SPSS Inc., Chicago, IL, USA). Data are expressed as mean ± SE and the level of significance was set at *p* ≤ 0.05.

## 5. Conclusions

Our results do not support racial differences in the expression of the TNFR1 complex or its signaling activity, particularly NF-κB activity, in this model. While HSS can mitigate some aspects of TNFR1 signaling similarly in ECs from both racial groups, its modulatory effect did not attenuate NF-κB binding activity. Given the higher burden of subclinical atherosclerosis in AA and the central role that TNF plays in atherogenesis, the TNFR1 signaling complex may still have a role in promoting racial differences through additional pathways not observed or measured in this study (e.g., IκB, A20, MAPK, or other novel associated factors). Therefore, we propose other aspects of TNFR1 signaling, such as MAP kinase signaling and their associated downstream effects, as recommended targets for future research.

## Data Availability

All data are provided in the manuscript, and further inquiries can be directed to the corresponding author.

## List of Abbreviations

TNF	Tumor necrosis factor
TNFR I	TNF receptor I
TRAF	TNFR-associated factor
NF-κB	Nuclear factor of kappa B
MAP Kinase	Mitogen-activated protein kinase
JNK	c-Jun N-terminal kinases
eNOS	Endothelial nitric oxide synthase
NO	Nitric oxide
sTNFR	Soluble TNF receptor
HSS	High laminar shear stress
AA	African American
ECs	Endothelial cells
CA	Caucasian American
CVD	Cardiovascular diseases
cIMT	Carotid intima-media thickness
FMD	Flow-mediated dilation
ET-1	Endothelin-1
HUVECs	Human umbilical vein endothelial cells
SOD1	Superoxide dismutase 1
IL-6	Interleukin-6
TACE	TNFα-converting enzyme
NIK	NF-κB-inducing kinase
cIAP1/2	Cellular inhibitor of apoptosis protein 1/2
AP-1	Activator protein 1
IL-1	Interleukin-1
VCAM-1	Vascular cell adhesion molecule 1
ICAM-1	Intracellular adhesion molecule 1
MCP-1	Monocyte chemoattractant protein 1
PBMCs	Peripheral blood mononuclear cells
